# Genetic and Protein Network Underlying the Convergence of Rett-Syndrome-like (RTT-L) Phenotype in Neurodevelopmental Disorders

**DOI:** 10.3390/cells12101437

**Published:** 2023-05-21

**Authors:** Eric Frankel, Avijit Podder, Megan Sharifi, Roshan Pillai, Newell Belnap, Keri Ramsey, Julius Dodson, Pooja Venugopal, Molly Brzezinski, Lorida Llaci, Brittany Gerald, Gabrielle Mills, Meredith Sanchez-Castillo, Chris D. Balak, Szabolcs Szelinger, Wayne M. Jepsen, Ashley L. Siniard, Ryan Richholt, Marcus Naymik, Isabelle Schrauwen, David W. Craig, Ignazio S. Piras, Matthew J. Huentelman, Nicholas J. Schork, Vinodh Narayanan, Sampathkumar Rangasamy

**Affiliations:** 1 Neurogenomics Division, Translational Genomics Research Institute (TGen), Phoenix, AZ 85004, USA; ericsf@stanford.edu (E.F.); msharifi@tgen.org (M.S.); rpillai@tgen.org (R.P.); nbelnap@tgen.org (N.B.); jdudson@tgen.org (J.D.); pvenugopla@tgen.org (P.V.); mbrzezinski@tgen.org (M.B.); lllaci@tgen.org (L.L.); bgerald@tgen.org (B.G.); gmills@tgen.org (G.M.); cbalak@tgen.org (C.D.B.); sszelinger@exactsciences.com (S.S.); wjepsen@tgen.org (W.M.J.); asiniard@illumina.com (A.L.S.); rrichholt@tgen.org (R.R.); mnaymik@tgen.org (M.N.); ipiras@tgen.org (I.S.P.); mhuentelman@tgen.org (M.J.H.); 2 Quantitative Medicine Division, Translational Genomics Research Institute (TGen), Phoenix, AZ 85004, USA; apodder@tgen.org (A.P.); nschork@tgen.org (N.J.S.); 3 Center for Rare Childhood Disorders (C4RCD), Translational Genomics Research Institute (TGen), Phoenix, AZ 85004, USA; kramsey@tgen.org (K.R.); msanchez-castillo@tgen.org (M.S.-C.); 4 Center for Statistical Genetics, Department of Neurology, Gertrude H. Sergievsky Center, Columbia University Medical Center, New York, NY 10032, USA; isabelle.schrauwen@gmail.com; 5 Department of Translational Genomics, University of Southern California, Los Angeles, CA 90033, USA; davidwcr@usc.edu; 6 City of Hope National Medical Center, Duarte, CA 91010, USA

**Keywords:** Rett syndrome, atypical RTT syndrome, Rett-syndrome-like phenotype, methyl-CpG-binding protein 2, neurodevelopmental disorders, overlapping phenotype, protein–protein interaction network

## Abstract

Mutations of the X-linked gene encoding methyl-CpG-binding protein 2 (*MECP2*) cause classical forms of Rett syndrome (RTT) in girls. A subset of patients who are recognized to have an overlapping neurological phenotype with RTT but are lacking a mutation in a gene that causes classical or atypical RTT can be described as having a ‘Rett-syndrome-like phenotype (RTT-L). Here, we report eight patients from our cohort diagnosed as having RTT-L who carry mutations in genes unrelated to RTT. We annotated the list of genes associated with RTT-L from our patient cohort, considered them in the light of peer-reviewed articles on the genetics of RTT-L, and constructed an integrated protein–protein interaction network (PPIN) consisting of 2871 interactions connecting 2192 neighboring proteins among RTT- and RTT-L-associated genes. Functional enrichment analysis of RTT and RTT-L genes identified a number of intuitive biological processes. We also identified transcription factors (TFs) whose binding sites are common across the set of RTT and RTT-L genes and appear as important regulatory motifs for them. Investigation of the most significant over-represented pathway analysis suggests that HDAC1 and CHD4 likely play a central role in the interactome between RTT and RTT-L genes.

## 1. Introduction

Rett syndrome (RTT), first described by Austrian pediatrician Andreas Rett, is a severe X-linked neurodevelopmental disorder mainly affecting females, which is characterized by the loss of spoken language and loss of purposeful hand use, as well as hand stereotypies that mimic handwashing [1,2,3]. Mutations of the X-linked gene encoding methyl-CpG-binding protein 2 (*MECP2*) cause RTT in girls, severe encephalopathy in male infants, and X-linked mental retardation [4]. Classical Rett syndrome is mostly attributed to *de novo* mutations in *MECP2*. Affected individuals display all the characteristic phenotypes, including the loss of acquired purposeful hand skills, loss of acquired spoken language, gait abnormalities, and stereotypic hand movements. Clinical criteria have been developed for patients with similar symptoms to RTT that lack one or another of the classical symptoms. These patients are referred to as having ‘atypical RTT syndrome’ [1,2,3]. Several pathogenic and likely causative variants for atypical RTT have been described, including the early seizure onset variant in the *CDKL5* (*cyclin-dependent kinase-like 5*) gene [5], a variant in the *MECP2* gene that leads to a unique preserved speech phenotype [6,7,8] rare variants in the *NTNG1* (*Netrin-G1)* gene [8], and a variant in the *FOXG1* (*Forkhead box G1*) gene [9].

Mutations that likely contribute to atypical RTT syndrome are found in genes besides *MECP2*, *CDKL5*, *NTNG1*, and *FOXG1*. However, most of the patients possessing these mutations display only some, but not all, the clinical features associated with classic and atypical RTT. These patients are referred to as having ‘Rett-syndrome-like phenotype (RTT-L)’ [10]. Given the genetic heterogeneity of RTT, atypical RTT, and RTT-L, it is likely that many of the genes and mutations that contribute to the phenotype overlap, whose identification could reveal additional molecular pathways and processes contributing to them.

We hypothesized that several of the mutated genes causing the RTT-L phenotype encode proteins that closely interact with known RTT and atypical RTT-associated proteins. We pursued an analysis of mutations and genetic variants resulting from a sequencing study of eight families with clinical features of RTT-L that did not possess mutations in the known RTT and atypical RTT genes—*MECP2*, *CDKL5*, *FOXG1*, and *NTNG1*. We then explored the PPIN between *MECP2*, *CDKL5*, *FOXG1*, and *NTNG1* and the genes harboring likely RTT-L mutations from our study. We also explored pathway enrichment analysis on our broad set of RTT and RTT-L genes.

## 2. Materials and Methods

### 2.1. Patient Samples

We identified 8 Caucasian trios whose offspring exhibited RTT-L clinical features according to the revised Rett Search International Consortium criteria and nomenclature [2]. Affected individuals did not possess mutations in the *MECP2*, *CDKL5*, *FOXG1*, and *NTNG1* genes. The Rett Search International Consortium criteria is an instrument for not only assessing and diagnosing RTT but also for classifying individuals into RTT and atypical RTT through a series of behavioral criteria. The parents of the affected children did not exhibit clinical features of RTT-L or intellectual disability. Genomic DNA for the sequencing studies from these trios was obtained from peripheral blood leukocytes. The study protocol and consent procedure were approved by the Western Institutional Review Board (WIRB; study number: 20120789). Informed consent was obtained from patients.

### 2.2. Sequencing

Genomic DNA was extracted from blood leukocytes for each member of the trios, and libraries were prepared using 1.2 g of DNA with the TruSeq DNA sample preparation and Exome Enrichment kit (62 Mb; Illumina, San Diego, CA, USA). Sequencing was performed on the Illumina platform and utilized several methods to identify potential pathogenic-disease-causing variants from the exome data following methods as previously described [11].

### 2.3. RTT-L Gene Literature Search

An extensive literature search was conducted on PubMed for peer-reviewed articles describing patients with RTT-L syndrome or RTT-syndrome-like disorder until June 2021 and manually curated a list of genes implicated in RTT-L [10,12,13,14,15,16,17,18,19,20,21,22,23,24,25,26,27,28,29,30,31,32,33,34,35,36,37,38,39,40,41,42,43,44]. We focused on genes where the literature clearly described the patient’s phenotypic features as overlapping with RTT syndrome. The list was meant to be exhaustive and was routinely updated. The genes identified from our sequencing were combined with the list of genes from the PubMed search. These characteristics were based on criteria outlined by the RettSearch International Consortium as well as frequently described proband phenotypes [2].

### 2.4. Physical Protein–Protein Interaction Networks (PPINs) for RTT- and RTT-L-Implicated Genes Network Analysis

The complete human protein interactome was downloaded from the Integrated Interactions Database (IID) [45]. The IID has information on protein–protein interaction (PPI) surveys associated with nine different curated databases (BioGRID, IntAct, I2D, MINT, InnateDB, DIP, HPRD, BIND, and BCI). The experimentally validated PPIs were further used to construct the resultant physical protein–protein interaction network (PPIN) in this study. The RTT-L-causing genes identified through our study and our exhaustive literature search, as well as the known RTT-implicated genes (MECP2, CDKL5, FOXG1, NTNG1), were used as the seed list for the PPIN construction. The network was further extended by including all the direct interacting protein partners of the RTT and RTT-L genes. To obtain a brain-specific PPIN for the RTT and RTT-L genes, we filtered all the PPIs based on their tissue-specific expression information in the human brain. The IID uses protein expression data sets from the Human Protein Atlas, whereby a protein is considered to be expressed in a tissue if its expression level is anything other than ‘not detected’ in the protein atlas database (Protein Atlas version 20.0 (Release date: 2020.11.19). The resultant network was visualized and further analyzed using the open-source software Cytoscape v3.9.1 [46]. To understand the topology of the overall networks, we computed metrics such as node degree (or connectivity), betweenness centrality (BC), and closeness centrality (CC). All the measurements describing the network topology were calculated using NetworkAnalyzer [47], which is a Java-based application (plugin) for Cytoscape. The first-order interacting protein partners for both the classical and atypical RTT genes were curated in a more sub-network-specific manner from the overall network. It is known that functionally related proteins are more connected than randomly chosen protein pairs [48]. The main idea was to identify a dense cluster (i.e., a strongly connected sub-graph) for the RTT genes in the overall PPIN. We performed sub-network searches in the overall using CytoHubba [49], which is another user-friendly interface for Cytoscape designed for identifying some of the more important nodes in biological networks based on network topology.

### 2.5. Functional Enrichments Analysis

Functional enrichment analysis was performed on our combined list of known and discovered RTT-L genes along with RTT- and atypical RTT-causing genes (RTT genes). A gene-set-based enrichment analysis was pursued using the g: GOSt tool from the g: Profiler website [50]. The functional annotation of the genes was obtained using the following data sources: biological process (BP) from Gene Ontology (GO); pathway information from KEGG, Reactome, and WikiPathways; and the regulatory motifs were searched using the transcription factors (TRANSFAC) and microRNA (miRTarBase) databases. Actual enrichment statistics and p-values were calculated by applying a two-sided hypergeometric test followed by a Bonferroni correction to the resulting p-values (*p* < 0.05) to identify enrichment or overrepresented terms in the respective databases [51].

## 3. Results

### 3.1. Clinical and Molecular Characterization of Pathogenic Variants

Here, we present a summary of the clinical reports for each RTT-L case in our study (Table 1). All eight patients were initially diagnosed as RTT or RTT-L by the clinician before the genetic diagnosis. Through WES, we identified likely *de novo* disease-causing mutations in *GABRG2* (patient 1), *GRIN1* (patient 2), *ATP1A2* (patient 3), *KCNQ2* (patient 4), *KCNB1* (patient 5), *GRIN2A* (patient 6), *TCF4* (patient 7), and *SEMA6B* (patient 8) genes, all of which are within evolutionarily conserved locations of the genes. In addition, all candidate mutations were not observed in controls in the Genome Aggregation Database (gnomAD). These mutations were in patients whose phenotypes overlap with those seen in patients with Rett syndrome (Table 1).

Patient 1 is heterozygous for a *de novo* substitution mutation (c.316G>A; p.Ala106Thr; CADD PHRED score: 22.2) in the *GABRG2* gene, resulting in a missense mutation in the ligand binding region of the encoded protein. This mutation was reported previously to be associated with childhood febrile seizures [52]. Patient 2 carries a heterozygous *de novo* mutation in the *GRIN1* gene, which is associated with neurodevelopmental disorders involving seizures [53,54]. The heterozygous c.2443G>C substitution resulted in a missense mutation (p. Gly815Arg; CADD PHRED score: 34). Patient 3 is heterozygous for a *de novo* substitution mutation (c.977T>G; p. Ile326Arg; CADD PHRED score: 26.2) in the *ATP1A2* gene, which is a private mutation. Analysis of patient 4’s exome revealed a de novo substitution mutation in the gene *KCNQ2* (c.740C>A; p.Ser247Ter; CADD PHRED score: 41)*,* which is associated with early infantile epileptic encephalopathy [30,55]. This variant is predicted to be targeted by nonsense-mediated decay (NMD). Patient 5 was identified as having a de novo missense variant in the *KCNB1* gene (c.916C>T; p.Arg306Cys; CADD PHRED score: 32), which is associated with early infantile epileptic encephalopathy [56]. Patient 6 is heterozygous for a de novo mutation (c.1845T>C) in the gene *GRIN2A*, which encodes a member of the glutamate-gated ion channel protein family and is associated with focal epilepsy and mental retardation [57]. This mutation results in missense (p. Asn614Ser; CADD PHRED score: 25.6) in exon 8 and affects the ligand-gated ion channel domain in the cytoplasmic region. Patient 7 is heterozygous for a de novo splice site mutation in the *TCF4* gene, and haploinsufficiency of the TCF4 (c.1486+5 G>T; CADD PHRED score: 23.4) causes rare Pitt–Hopkins syndrome [58]. The WES of Patient 8 revealed the presence of a de novo frameshift mutation in the gene *SEMA6B*, predicted to cause a premature truncated protein (c.1991delG; p.Gly664fsX21; CADD PHRED score: 16.8), and de novo heterozygous mutations in *SEMA6b* is indicated to cause progressive myoclonic epilepsy-11 [59].

### 3.2. Curation of Gene Lists

After observing phenotypic overlap between features of RTT and our patients, we curated a list of genes implicated in causing RTT-L from articles in peer-reviewed journals. Through an exhaustive literature search, 58 genes were identified (Supplementary Table S1) as harboring *de novo* damaging or chromosomal deletion mutations contributing, if not likely causing, RTT-L [10,12,13,14,15,16,17,18,19,20,21,22,23,24,25,26,27,28,29,30,31,32,33,34,35,36,37,38,39,40,41,42,43,44]. In addition to the curated list, we included the genes *KCNQ2, GABRG2, GRIN1, ATP1A2, KCNB1, GRIN2A, TCF4,* and *SEMA6B* identified from our cohort to the RTT-L list and added classical RTT-causing genes such as *MECP2* and atypical *RTT* genes, such as *CDKL5, FOXG1,* and *NTNG1*, in the curated gene list table for analysis (Table 2).

### 3.3. Phenotype Clustering of RTT-L Patients

We analyzed the phenotypes of RTT-L patients to evaluate the frequency of main and supportive RTT criteria that appeared in our patients. Of the main criteria, RTT-L patients often displayed at least two of the following clinical RTT features: regression, partial or complete loss of acquired purposeful hand skills, partial or complete loss of acquired spoken language, gait abnormalities, and hand stereotypies identified through the phenotype clustering of the RTT-L patients (Supplementary Figure S1).

### 3.4. Protein–Protein Interaction Networks (PPINs) across RTT and RTT-L Genes

**Figure 1:**
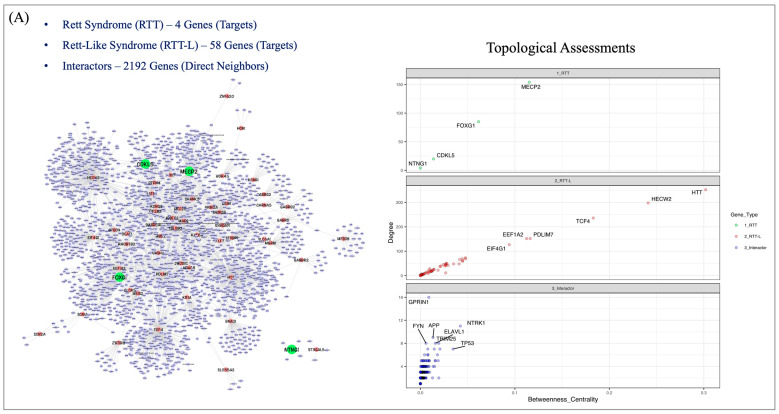

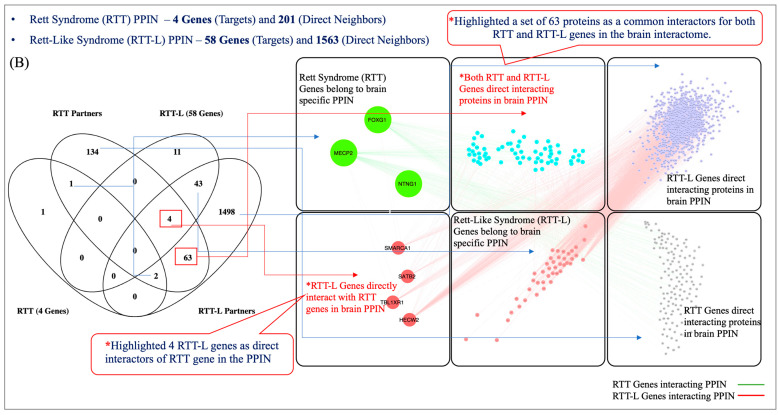
RTT and RTT-L genes connecting PPIN: (**A**) The experimental protein–protein interactions (PPIs) (in general—non-tissue-specific) of 4 RTT and 58 RTT-L genes together identified 2192 interacting partners in human interactome. The topological assessments show the degree (connectivity) and betweenness centrality distribution (box plot) of the genes and highlighted the hub-like RTT, RTT-L, and interactor genes with higher degree of connectivity in the PPIN. Genes such as NTNG1 (RTT) and ST3GAL5 (RTT-L) are not closely connected with the main network but share distinct set of interactions with other proteins in human interactome. (**B**) A physical protein–protein interaction (PPI) graph of 4 RTT and 58 RTT-L genes together identified 201 and 1563 direct interacting partners, respectively, in the human-brain-specific network. The Venn diagram highlights the common interactors present in both RTT and RTT-L genes’ specific PPINs. RTT—Rett syndrome and RTT-L—Rett-like Syndrome.

Since we observed phenotypic overlap between clinical features of RTT and the RTT-L patients in our cohorts, we were motivated to investigate protein–protein interactions (PPIs) involving genes implicated in RTT-L syndrome and the genes we identified from our sequencing study (Table 2). We used the PPI data sets from the Integrated Interactions Database (IID) to identify experimentally validated protein partners for both RTT and RTT-L genes. We found that a total of 2196 proteins directly interact with both the RTT and RTT-L genes in the human protein interactome. Together, 2871 interactions were mapped between RTT, RTT-L, and 2192 neighboring proteins. The overall physical protein–protein interaction network (PPIN) for RTT-L syndrome is shown in Figure 1A. The topological arrangements of the proteins were further analyzed using different network centrality parameters, such as the node degree and betweenness centrality, to identify the hub-like proteins in the constructed PPIN (Supplementary Table S2). The centrality comparison between three different groups of proteins (RTT, RTT-L, and other interactors) highlighted several important nodes that are highly connected and, thus, might play an important regulatory role in the overall PPIN (Figure 1A).

RTT-L genes such as HTT, HECW2, and TCF4 show very high levels of connectivity compared to RTT genes such as MECP2, FOXG1, and CDKL5 in the overall network. One of the RTT genes, NTNG1, exhibits a very distinct set of interactions and does not crosstalk with the main PPIN. We also noticed that NTNG1 only interacts with four different proteins (HSPA6, LRRC4, LRRC4C, and GAS6) and maintains a distinct set of interactions in the constructed PPIN. Our analysis also identified several neighboring protein partners for both RTT and RTT-L genes that show a high level of connectivity in the overall network. For example, GPR1N1, FYN, APP, and NTRK1 show enhanced interactions and appear as key neighboring protein partners in the constructed PPIN. 

The topological arrangements of all four RTT genes in the constructed PPIN were further investigated using sub-network analysis. We explored two particular sub-networks for this study—The first one is the MECP2 (typical) gene mediated sub-network (Supplementary Figure S2A), where we found a close contact between MECP2 and another RTT gene, CDKL5. Two RTT-L genes (HECW2 and TBL1XR1) also show a direct connection with MECP2 in the same sub-network. Apart from that, 151 other proteins share a common set of interactions in the MECP2 mediated sub-network. We also included all four RTT genes, i.e., classical and atypical, together and constructed another sub-network (Supplementary Figure S2B). We found a total of four RTT-L genes (HECW2, TBL1XR1, SMARCA1 and SATB2) share direct connectivity with MECP2, CDKL5 and FOXG1 and play an important role in maintaining the cross-talk between RTT genes in the overall PPIN. Altogether, 255 other proteins along with 4 RTT and 4 RTT-L genes show proximity in the RTT gene sub-network.

To understand the tissue-specific connectivity of RTT and RTT-L genes, we further restricted our PPIN analysis to human brain tissue. The existing PPIN was refined based on brain-specific expression profiling of the proteins. A physical protein–protein interaction (PPI) graph of RTT and RTT-L genes together identified 201 and 1563 direct interacting partners, respectively, in the human-brain-specific network (Figure 1B). We highlighted a total of 63 protein partners, which share a common interaction with both RTT and RTT-L genes and are, therefore, important for inter-network communication in the human-brain-specific PPIN (Supplementary Table S3). We also observed that the RTT gene CDKL5 has lost its connectivity in the brain-specific PPIN due to inadequate protein level expression information in the human brain.

### 3.5. Functional Enrichment

**Figure 2:**
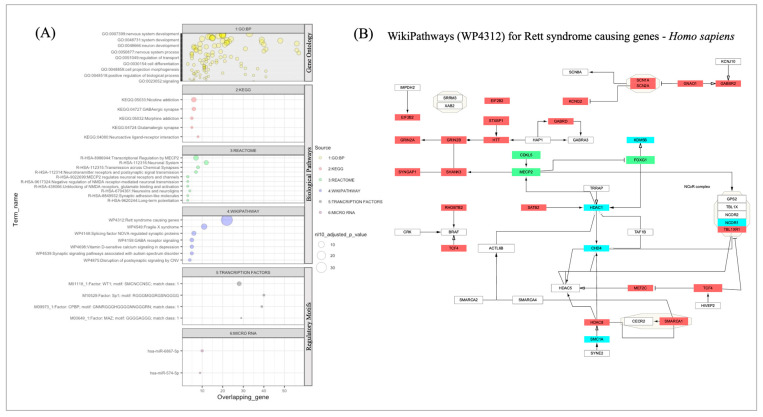
Functional Enrichment Analysis for RTT and RTT-L Genes: (**A**) A gene-set-based enrichment analysis results for RTT (N1 = 4) and RTT-L (N2 = 58) genes together using the Gene Ontology (biological process), pathway databases (KEGG, REACTOME, and WIKIPATHWAYS) and regulatory motifs databases (transcription factors and microRNA). The X-axis represents the number of overlapping genes from our given gene sets in each enrichment term. The dot size highlights the enrichment statistics (*p*-values) for each term, and the dot color represents the source of each term in the respective databases. (**B**) The RTT and RTT-L genes are highlighted in one of the well-documented WikiPathways (WP4312) for Rett-syndrome-causing genes—Homo sapiens. We identified a total of 5 genes (highlighted in cyan color) that are part of the PPIN, which share common interaction with both RTT and RTT-L genes (as shown in Figure 1A). Genes such as HDAC1 and CHD4 play a central role in the underline pathway.

We then conducted functional profiling using a gene-set-based enrichment analysis on the complete RTT and RTT-L gene lists to identify significantly altered biological processes and pathways among the genes (Figure 2A). The biological functions identified (adjusted *p* < 0.05) were ranked according to the *p*-values for an enrichment test (Supplementary Table S4). The RTT and RTT-L genes together showed a significant over-representation of GO biological processes involving nervous system development, chemical synaptic transmission, behavior, cognition, and learning or memory processes. Similarly, the gene sets together also had an over-representation of the biological pathways including GABAergic and glutamatergic synapse (KEGG), transcriptional regulation by MECP2 and neuronal system (Reactome), and Rett-syndrome-causing genes and fragile X syndrome (WikiPathways).

We also identified four different transcription factors (TFs)—WT1, Sp1, CPBP, and MAZ—and two micro-RNA (microRNA)—hsa-miR-6867-5p and hsa-miR-574-5p—of which the binding sites are conserved across the gene set and appear as important regulatory motifs for the RTT and RTT-L genes. 

We further investigated the most significant over-represented pathway in our analysis, i.e., one of the well-documented MECP2 regulatory WikiPathways (WP4312) for Rett-syndrome-causing genes in Homo sapiens (adjusted *p*-value = 5.1 × 10^−37^), and identified a total of 3 RTT genes and 22 RTT-L genes, together representing the following pathway. We also mapped the RTT and RTT-L genes’ neighboring protein partners from WP4312 pathway connectome and highlighted a total of five proteins (KDM5B, HDAC1, CHD4, NCOR1, and SMC1A) that are part of the pathway and shared a common interaction with both RTT and RTT-L genes in our constructed PPIN (as shown in Figure 2B). HDAC1 and CHD4 seem to play a central regulatory role in the underlining pathway. MECP2 activates the formation of the MECP2-HDAC complex, which, in turn, inhibits MEF2C, another RTT-L gene. Additionally, we found that two transcription factors—STAB2 and TCF4—are part of the RTT-L genes and might play a regulatory role in the pathway by altering FOXG1 expression.

## 4. Discussion

The application of WES to patients with features of RTT resulted in the identification of several likely causative mutations beyond those found in *MECP2*, *CDKL5*, *FOXG1*, and *NTNG1*. Patients with classical and atypical RTT display characteristic phenotypes that are well defined. However, there are many clinical diagnoses in settings in which individuals exhibit some, but not all, phenotypes associated with RTT, raising the question about overlap in the genetic determinants of RTT and RTT-like (RTT-L) clinical phenotypes. To characterize the potential overlap between RTT-associated genes and RTT-L-associated genes, we pursued a whole exome sequencing (WES) study of eight trios with offspring with the RTT-L phenotype and combined the genes we found to be associated with RTT-L with known RTT genes for protein–protein interaction network (PPIN) and pathway enrichment analyses. WES analysis of the eight trios led to the identification of unique de novo candidate RTT-L mutations in genes known to cause childhood epilepsy (*KCNB1*, *KCNQ2*, and *GABRG*) [52,60,61,62], mental retardation (*GRIN1* and *GRIN2A*) [63,64], hemiplegia, developmental and epileptic encephalopathy, and microcephaly (*ATP1A2*) [65,66,67].

A combined analysis of RTT-L-associated genes from our WES study and known RTT genes suggests shared neurological processes and pathways contribute to RTT-L features and likely interact with the RTT-implicated genes *MECP2*, *CDKL5*, *FOXG1*, and *NTNG1*. Examples of functional overlap of the function of the genes associated with RTT and RTT-L include the potassium voltage-gated channel subfamily B member 1 (*KCNB1*) gene, which is responsible for transmembrane potassium transport in excitable membranes, and potassium voltage-gated channel subfamily Q member 2 (*KCNQ2*), which is responsible for the subthreshold electrical excitability of neurons, as well as the responsiveness to synaptic inputs. These transmembrane ion proteins are functionally relevant in the context of RTT and RTT-L, with *KCNB1* implicated in earlier studies of RTT-L [68,69]. Other examples of the functional overlap of the genes are glutamate ionotropic receptor NMDA type subunit 1 (*GRIN1*) and glutamate ionotropic receptor NMDA type subunit 2A (*GRIN2A*), both of which are essential in excitatory neurotransmission, verbal memory, and cognitive function, probably through regulating the patterning of neuron dendritic arborizations [63,64,70,71,72,73], which is similar to the impacts caused due to RTT mutations. ATP1A2 mutations cause variable phenotypes such as hemiplegia, epilepsy, and intellectual disability [65,66,67,74]. We found a patient with the RTT-L phenotype carrying the private potential pathogenic mutation in the ATP1P2 gene that encodes the alpha2 isoform of the Na(+), K(+)-ATPase. A mutation in Na(+), K(+)-ATPase has been shown to share many clinical features of RTT [75,76], and reduced neuronal activity was found to be a distinct abnormality of Rett syndrome neurons in human and preclinical models [77,78,79]. Thus, the ATP1A2 mutation may contribute to the pathogenesis of RTT-L linked to its molecular association with MECP2. Transcription factor 4 (TCF4) is a primary helix–loop–helix transcription factor that plays an essential role in neural development. Mutations in the *TCF4* gene cause Pitt–Hopkins syndrome, a rare neurological disorder characterized by developmental delay and intellectual disability [80,81]. Patients with *TCF4* mutations present with phenotype overlap with Rett syndromes and are often diagnosed as RTT-L [29,82]. Pathogenic variants in genes encoding Gamma-aminobutyric acid type A receptor gamma2 subunit (*GABRG2*) were first identified to cause developmental disorders characterized by the classical Rett syndrome phenotype [20]. It is interesting to note that GABA-receptor-mediated neurotransmission is a hallmark of several Rett syndrome phenotypes in animal and in vitro *MECP2* mutant model systems [83]. Additionally, the restoration of *Mecp2* expression in GABAergic neurons rescues features of Rett syndrome in a mouse model [84]. These findings highlight the critical role of GABAergic neurons in the RTT-L phenotype. Semaphorins, a significant player in axon guidance, are involved in peripheral and central nervous system development. De novo pathogenic variants in *SEMA6B* were identified to cause progressive myoclonic epilepsy-11 and have also been described as causing RTT-like clinical phenotypes [10]. Overall, these findings from the well-characterized patient phenotypes from our cohort indicate a significant degree of overlap between the genetic basis of RTT-L and other neurodevelopmental disorders.

Functional enrichment analysis of an expanded list of RTT and RTT-L genes further demonstrates the genetic diversity of the clinical RTT phenotype. Past studies have noted that genes causing RTT-L are involved in neurodevelopmental diseases such as Dravet syndrome (*SCN1A*), Pitt–Hopkins syndrome (*TCF4*), and Huntington’s disease (*HTT*) [10,17,68]. This clinical overlap with similar neurodevelopmental diseases suggests the presence of shared functional networks among diseases with similar, but not identical phenotypic manifestations, suggesting a more detailed study of RTT and RTT-L associated genes could reveal functional overlap. Network analysis of the PPI interaction network generated with the RTT- and RTT-L genes suggests significantly interconnected protein network implicating genes with high betweenness centrality (the number of shortest paths in the network that pass through the gene). Protein–protein interaction networks (PPINs) across RTT and RTT-L genes analysis indicate RTT-L genes, such as HTT, HECW2, and TCF4, exhibit very high levels of connectivity compared to MECP2, FOXG1, and CDKL5 in the overall network. Proteins such as GPR1N1, FYN, APP, and NTRK1 show enhanced interactions and appear as key neighboring protein partners in the constructed PPIN. Interestingly, HECW2, TBL1XR1, SMARCA1, and SATB2 were identified to have direct connectivity with MECP2, CDKL5, and FOXG1 and play an important role in maintaining the crosstalk between RTT genes in the overall PPIN. These genes likely serve as key interactors with genes that cause RTT-L syndrome.

RTT-L-causing genes are directly involved in the *MECP2*-mediated pathways of chromatin regulation (*HDAC2*, *MEF2C*, *CREB1*, and *GRIN1*), upregulation of glutamate, and downregulation of dopamine and GABA pathways (*CREB1*, *KCNA2*, *GRIN1*, *KCNB1*, *GRIN2A*, *GRIN2B*, and *KCNQ2*) [85]. A pathway analysis of RTT and RTT-L genes also showed enrichment of GO biological processes involving nervous system development, chemical synaptic transmission, behavior, cognition, and learning or memory processes [86,87]. The involvement of many RTT-L-implicated genes in *MECP2* function indicates that the replication of many of the clinical features of RTT-L like that of classical or atypical RTT could be attributed to the disruption of one of *MECP2*’s transcription regulatory activities. We found two transcription factors—SATB2 and TCF4—might play a regulatory role in the pathway by altering FOXG1 expression. This interaction between *MECP2* and RTT-L implicated genes reinforces the notion that there is not a unique biological process or molecular pathway involved in RTT that is disrupted; rather, because *MECP2* is pleiotropic and involved in a variety of pathways by controlling transcription, and as seen from Erhart et al., *MECP2* functionality can result in alterations in the many different pathways it controls [85].

Based on the characteristics of our patient cohort and the other diseases with which the RTT-L syndrome genes we identified are also associated, it is intuitive that RTT-L patients tend to have clinical features ranging from (most to least frequent) epilepsy, intellectual disorders, regression, microcephaly, to hand stereotypies (Table 1). Our analysis of RTT and RTT-L clinical phenotype clustering suggests that many different combinations of clinical features could aid in the delineation of central phenotypes associated with RTT-L, as well as guide diagnoses for genetic testing in future patients. Further genetic characterizations of RTT-L patients are critical for future genotype–phenotype correlations and the refinement of a set of core RTT-L clinical features. Finally, the characterization of the biological processes and pathways implicated in RTT-L-syndrome-causing genes suggests common HDAC and CHD4 pathways that could be a focus of future targeted therapies. HDAC is already considered a potential target for Rett syndrome therapy [88]; thus, there is great potential in systematically exploring the role of HDAC and CHD4 in developing novel therapies. Altogether, the analysis of PPI networks of RTT-L-causing genes identifies novel genes of high centrality that could be future candidate genes in RTT-L syndrome.

## Figures and Tables

**Table 1:** Clinical and genetic characterization of RTT-L cohort.

Patient	Developmental Regression	Developmental Delay	Intellectual Disability	Microcephaly	Loss of Hand Use	Stereotyped Hand Movements	Involuntary Tongue Movements	Hyperventilation	Choreoathetosis	Early Epileptic Encephalopathy	Hypotonia	Scoliosis	*Gene*	Protein	Variant: Genomic Coordinates	cDNA Change	Protein Change	Gene-Diseases Association	CADD PHRED Score
1	Y	Y	Y	Y	N	N	N	N	N	Y	Y	N	*GABRG2*	Gamma-Aminobutyric Acid Type A Receptor Gamma2 Subunit	5:161522557	c.316 G>A	p.Ala106Thr	Epilepsy, generalized, with febrile seizures plus, type 3	22.2
2	N	Y	Y	Y	N	N	N	Y	Y	N	Y	N	*GRIN1*	Glutamate Ionotropic Receptor NMDA Type Subunit 1	9:140058120	c.2443 G>C	p.Gly815Arg	Mental retardation, autosomal dominant 8	34
3	Y	Y	Y	Y	Y	Y	N	N	Y	Y	Y	N	*ATP1A2*	ATPase Na+/K+ Transporting Subunit Alpha 2	1:160097570	c.977 T>G	p.Ile326Arg	Alternating hemiplegia of childhood	26.2
4	N	Y	Y	N	N	N	N	N	Y	Y	N	N	*KCNQ2*	Potassium Voltage-Gated Channel Subfamily Q Member 2	20:63442482	c.740 C>A	p.Ser247Ter	Epileptic encephalopathy, early infantile, 7	41
5	Y	Y	Y	N	N	N	N	N	N	N	N	N	*KCNB1*	Potassium Voltage-Gated Channel Subfamily B Member 1	20:47991181	c.916 C>T	p.Arg306Cys	Epileptic encephalopathy, early infantile, 26	32
6	N	Y	N	N	N	Y	N	N	Y	N	N	Y	*GRIN2A*	Glutamate Ionotropic Receptor NMDA Type Subunit 2A	16:9923446	c.1845 T>C	p.Asn614Ser	Epilepsy, focal, with speech disorder and with or without mental retardation	25.6
7	Y	Y	Y	Y	Y	Y	Y	Y	N	N	Y	N	*TCF4*	Transcription Factor 4	chr18:52901774	c.1486+5 G>T	IVS16+5 G>T (Splice Variant)	Pitt-Hopkins	23.4
8	N	Y	Y	Y	N	Y	N	N	N	N	Y	N	*SEMA6B*	Semaphorin 6B	chr19:4544288	c.1991delG	p.G664AfsX21	Autism Spectrum Disorder	16.8

Y = Yes/N = No.

**Table 2:** List of genes contributing to classical RTT, atypical RTT, RTT-L features of our cohort and those identified from literature.

Genes List
RTT Syndrome (Classical): *MECP2*
RTT Syndrome (Atypical): *MECP2*, *CDKL5*, *FOXG1*, *NTNG1*
RTT-L Genes Identified at C4RCD: *KCNQ2*, *GABRG2*, *GRIN1*, *ATP1A2*, *KCNB1*, *GRIN2A*, *TCF4*, *SEMA6B*
RTT-L List (Literature): *ADAM23*, *AGAP6*, *ANKRD31*, *BTBD9*, *CHRNA5*, *CLCN5*, *CREB1*, *DEAF1*, *EEF1A2*, *EIF2B2*, *EIF4G1*, *GABBR2*, *GABRG2*, *GABRB2*, *GABRD*, *GNAO1*, *GRIN1*, *GRIN2A*, *GRIN2B*, *HCN1*, *HDAC8*, *HECW2*, *HTT*, *IQSEC2*, *JMJD1C*, *KAT6A*, *KCNB1*, *KCNQ2*, *KIF1A*, *KLF7*, *MAP2*, *MBD2*, *MEF2C*, *MEIS2*, *MFSD8*, *MGRN1*, *PDLIM7*, *PTPN4*, *RHOBTB2*, *SATB2*, *SCN1A*, *SCN2A*, *SCN8A*, *SHANK3*, *SHROOM4*, *SLC35A2*, *SLC6A1*, *SMARCA1*, *ST3GAL5*, *STXBP1*, *SYNGAP1*, *TBL1XR1*, *TCF4*, *VASH2*, *WDR45*, *ZFX*, *ZNF238*, *ZNF620*

## Data Availability

No data are presented. The figures are original.

## Supplementary Materials

The following supporting information can be downloaded at: https://www.mdpi.com/article/10.3390/cells12101437/s1. Table S1: Curated RTT-L Gene List. Table S2: The centrality values (degree and betweenness) for genes in RTT and RTT-L connecting. Table S3: RTT and RTT-L genes connecting protein partners in human-brain-specific PPIN. Table S4: Functional Enrichment Analysis for RTT and RTT-L Genes. Figure S1: Phenotype clustering of RTT-L patients from the study cohort. Figure S2: RTT-Gene-Associated Sub-Network—Classical and Atypical.
