# Supplementary material for: Genetic and Protein Network Underlying the Convergence of Rett-Syndrome-like (RTT-L) Phenotype in Neurodevelopmental Disorders
Source: Cells. 2023 May 21;12(10):1437. doi: 10.3390/cells12101437 (PMC10217403; doi:10.3390/cells12101437)
Supplement: Supplementary file 1 [file cells-12-01437-s001.zip › Supplement Table S1.pdf]

Supplement Table S1 : Curated RTT-L Gene List

RTT-L Gene List	References
ADAM23	[12]
AGAP6	[10]
ANKRD31	[10]
BTBD9	[10]
CHRNA5	[13,40]
CLCN5	[14]
CREB1	[12]
DEAF1	[15]
EEF1A2	[16] [17]
EIF2B2	[17]
EIF4G1	[17]
GABBR2	[17] [18]
GABRD	[19]
GABRG2	[20]
GNAO1	[21]
GRIN1	[44]
GRIN2A	[22
GRIN2B	[10]
HCN1	[10, 23]
HDAC8	[24]
HECW2	[25]
HTT	[17]
IQSEC2	[26]
JMJD1C	[27]
KAT6A	[28]
KCNB1	[29]
KCNQ2	[30]
KIF1A	[44]
KLF7	[12]
MAP2	[12]
MBD2	[31]
MEF2C	[32]
MEIS2	[29]
MFSD8	[33]
MGRN1	[10]
PDLIM7	[10]
PTPN4	[34]
RHOBTB2	[17]
SATB2	[35

SCN1A	[10]
SCN2A	[36]
SCN8A	[37]
SHANK3	[38]
SHROOM4	[17]
SLC35A2	[17]
SLC6A1	[10]
SMARCA1	[17]
ST3GAL5	[39]
STXBP1	[17]
SYNGAP1	[40,41]
TBL1XR1	[42]
TCF4	[17]
VASH2	[10]
WDR45	[43]
ZFX	[17]
ZNF238	[17]
ZNF620	[10]
GABRB2	[20]
