# Supplementary figures and images for: Genetic and Protein Network Underlying the Convergence of Rett-Syndrome-like (RTT-L) Phenotype in Neurodevelopmental Disorders
Source: Cells. 2023 May 21;12(10):1437. doi: 10.3390/cells12101437 (PMC10217403; doi:10.3390/cells12101437)

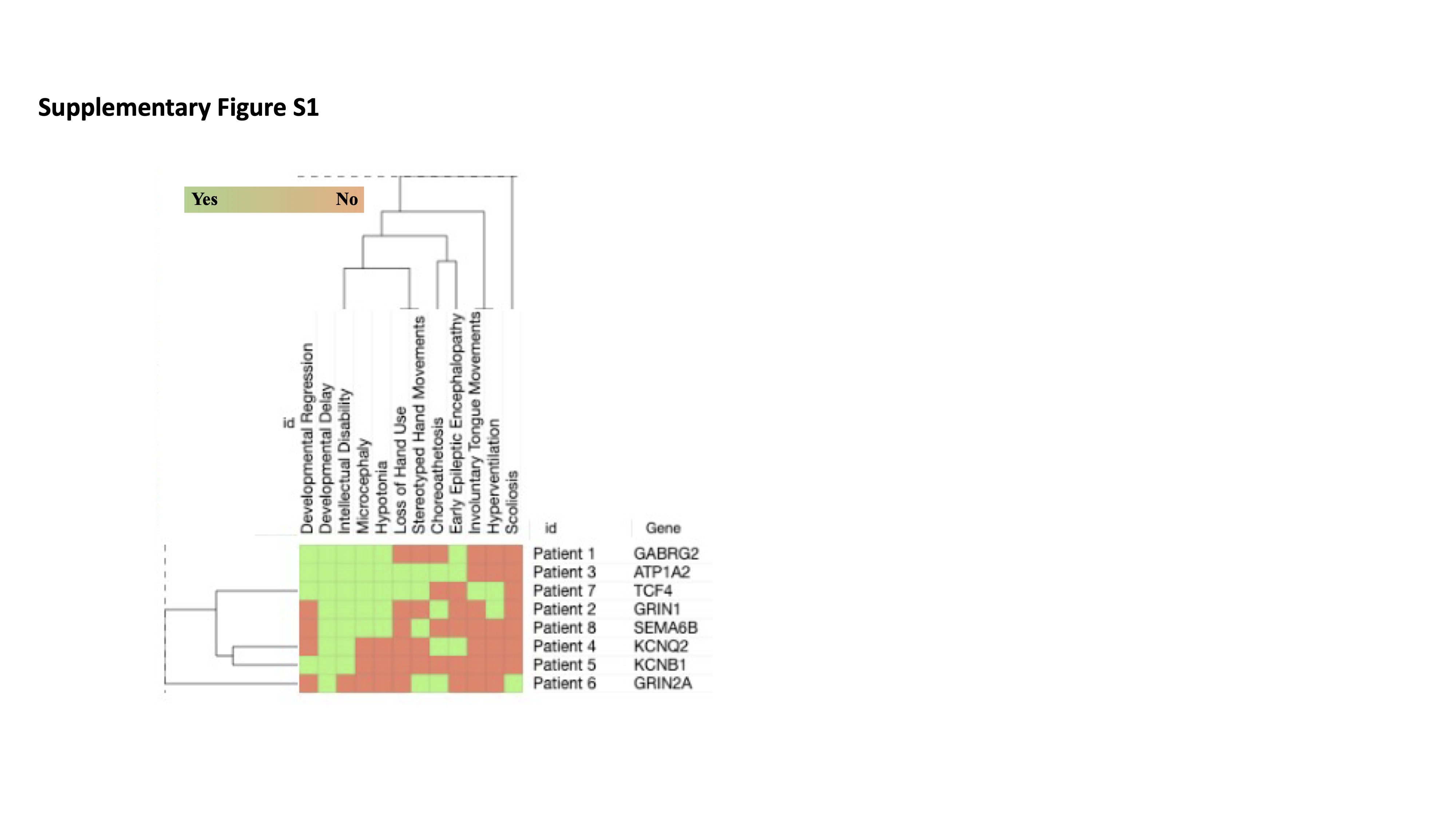

Supplement: Supplementary file 1 [file cells-12-01437-s001.zip › Supplementary Figure S1.tiff]

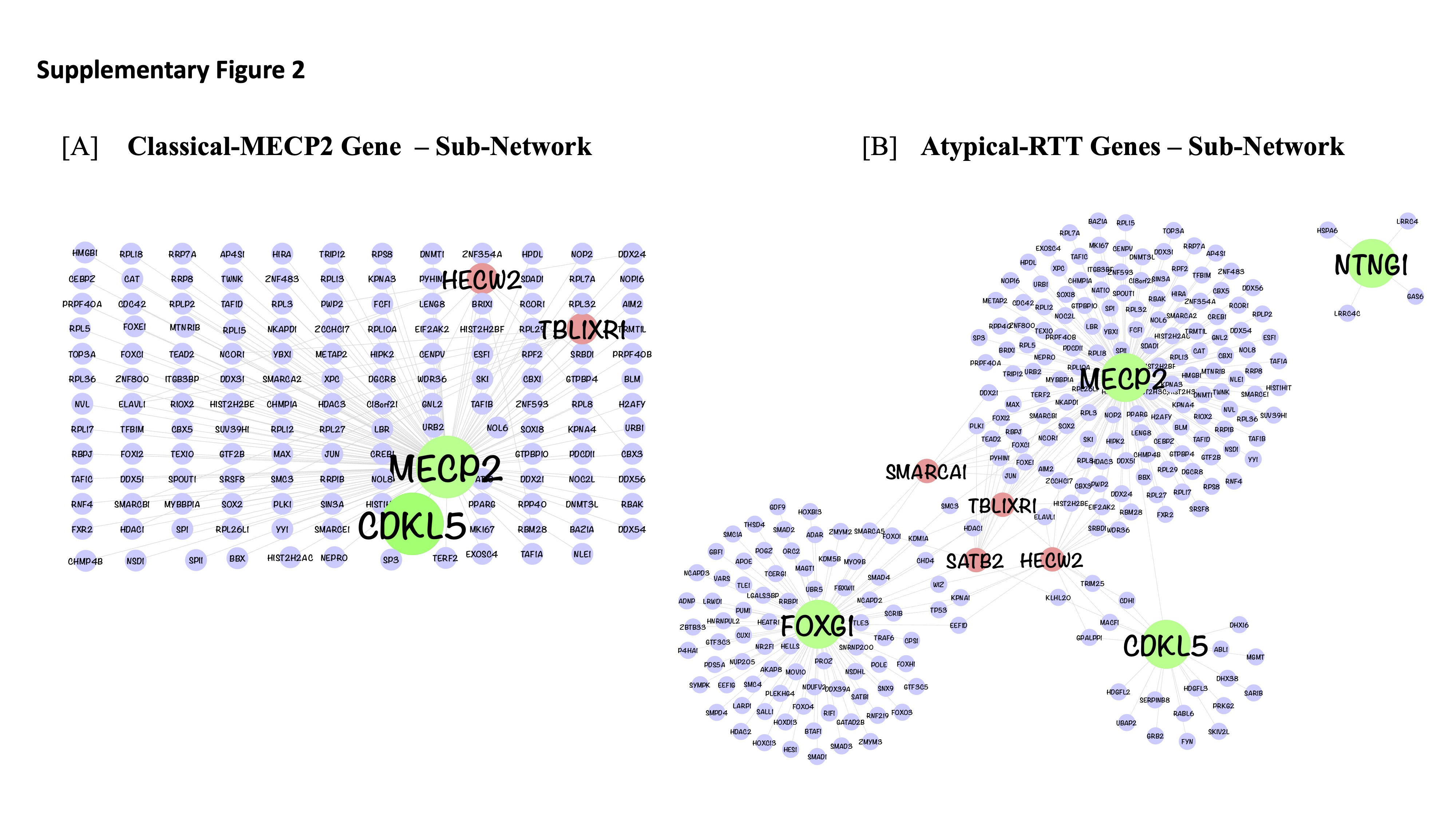

Supplement: Supplementary file 1 [file cells-12-01437-s001.zip › Supplementary Figure S2.tiff]
